# The Circadian Response of Intrinsically Photosensitive Retinal Ganglion Cells

**DOI:** 10.1371/journal.pone.0017860

**Published:** 2011-03-14

**Authors:** Andrew J. Zele, Beatrix Feigl, Simon S. Smith, Emma L. Markwell

**Affiliations:** 1 Institute of Health and Biomedical Innovation, Queensland University of Technology, Brisbane, Australia; 2 School of Optometry, Queensland University of Technology, Brisbane, Australia; 3 Centre for Accident Research and Road Safety Queensland, Queensland University of Technology, Brisbane, Australia; University of Houston, United States of America

## Abstract

Intrinsically photosensitive retinal ganglion cells (ipRGC) signal environmental light level to the central circadian clock and contribute to the pupil light reflex. It is unknown if ipRGC activity is subject to extrinsic (central) or intrinsic (retinal) network-mediated circadian modulation during light entrainment and phase shifting. Eleven younger persons (18–30 years) with no ophthalmological, medical or sleep disorders participated. The activity of the inner (ipRGC) and outer retina (cone photoreceptors) was assessed hourly using the pupil light reflex during a 24 h period of constant environmental illumination (10 lux). Exogenous circadian cues of activity, sleep, posture, caffeine, ambient temperature, caloric intake and ambient illumination were controlled. Dim-light melatonin onset (DLMO) was determined from salivary melatonin assay at hourly intervals, and participant melatonin onset values were set to 14 h to adjust clock time to circadian time. Here we demonstrate in humans that the ipRGC controlled post-illumination pupil response has a circadian rhythm independent of external light cues. This circadian variation precedes melatonin onset and the minimum ipRGC driven pupil response occurs post melatonin onset. Outer retinal photoreceptor contributions to the inner retinal ipRGC driven post-illumination pupil response also show circadian variation whereas direct outer retinal cone inputs to the pupil light reflex do not, indicating that intrinsically photosensitive (melanopsin) retinal ganglion cells mediate this circadian variation.

## Introduction

Intrinsically photosensitive (melanopsin) retinal ganglion cells (ipRGCs) provide irradiance input to the suprachiasmatic nucleus (SCN), and also act as a relay for extrinsic dark and light signals from the rod and cone photoreceptors to the SCN [1], [2], [3], [4], [5]. IpRGCs express the photopigment melanopsin and mediate non-image forming photoreception [6]. Their input synchronizes the SCN to the solar day that maintains the human circadian rhythm near a 24 hour cycle by driving nocturnal synthesis of the pineal hormone melatonin and feedback loops to mediate clock information to the peripheral tissues and induce circadian phase and sleep. The electrophysiological activity of the SCN shows a circadian rhythm with a morning and evening peak in mammals *in vitro* [7], [8]. In humans, SCN activity measured via blood melatonin suppression in response to monochromatic and polychromatic light also changes absolute sensitivity to photic stimulation during the night [7], [9]. This, together with an increase in pupil constriction during the night, suggests a temporal change in the spectral sensitivity of circadian phototransduction [9] that may be measured directly via the pupil light reflex.

The pupil light reflex is an objective measure of visual and pupillary pathways. Outer retinal rod and cone photoreceptor inputs control the initial light constriction of the pupil [10], [11], [12] and the reported circadian characteristics of the pupil light reflex driven by the outer retina are inconsistent [13], [14], [15], [16], [17]. Visual function and retinal processing also undergo rhythmic variation [18], [19] and melanopsin is involved in this regulation [20], [21]. The post-illumination pupil response (PIPR) [22], [23] is an intrinsic ipRGC controlled [20], [24], [25] constriction that is sustained for >30 seconds after light offset when the pupil redilates. *In vitro* ipRGC recordings in macaque and human retina display a typical transient increase in firing rate at stimulus onset and a unique sustained firing that continues after light offset [1]. This sustained, intrinsic ipRGC photoresponse after light offset controls the post-illumination pupil response [23]. The ipRGC-mediated PIPR is a robust pupil function that can be reliably derived and reproduced in normal persons [10], [11], [26]. Whether or not it undergoes circadian variation has not been tested.

In addition to their intrinsic response, inner retinal ipRGCs receive inputs from outer retinal rod and cone photoreceptors [1], [11], [27]. The intrinsic ipRGC response amplitude and time-to-peak increase with irradiance [1], [28] and the total number of spikes during the sustained depolarization after light offset is linearly proportional to retinal irradiance in the photopic range between about 11.5 and 14.7 log photons.cm^−2^.s^−1^ [1], [29]. This light evoked output is used for circadian photoentrainment, but it is unknown if central mechanisms attenuate this output. *In vitro* electrophysiological recordings of rat retina suggest that ipRGCs lack autonomous circadian modulation of sensitivity [30]. However, if ipRGC sensitivity is extrinsically regulated by central mechanisms, the *in vivo* functional ipRGC response measured under constant exogenous circadian cues and environmental illuminations may explicate any extrinsic circadian dependent variation in ipRGC sensitivity.

The present study measured the direct functional contribution of ipRGCs to the pupil light reflex in humans to determine the diurnal response of ipRGCs and the effect of central gating on ipRGC sensitivity. To control exogenous circadian cues, a 24 h, constant routine laboratory protocol was implemented so that the presence of endogenous rhythms could then be detected. The diurnal contribution of outer retinal (cone photoreceptors) and inner retinal (intrinsic ipRGC response, cone inputs to ipRGCs) inputs to the pupil light reflex was isolated and their phase position to the central circadian rhythm was expressed as a function of salivary melatonin concentration.

## Results

### Outer retinal contributions to the pupil light reflex do not show diurnal variation

**Figure 1:**
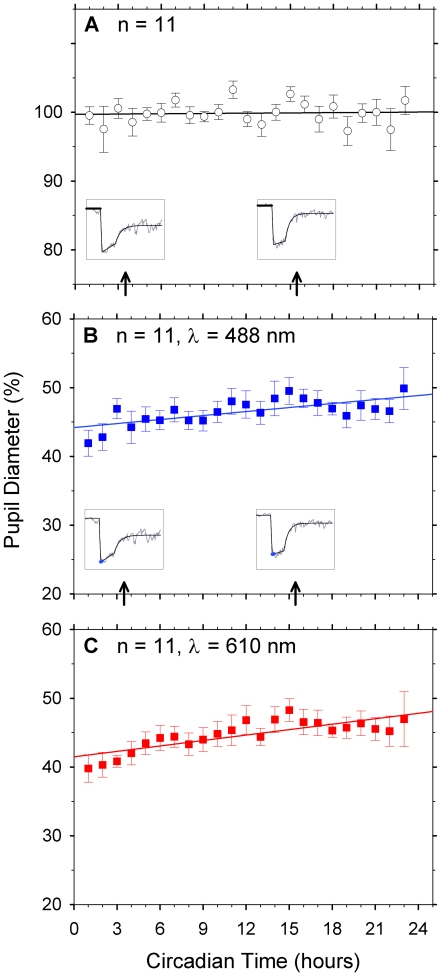
Diurnal cone photoreceptor contributions to the human pupil light reflex. (A) Baseline pupil diameter of 11 participants (mean ± s.e.m) viewing a uniform photopic screen, recorded over 20–24 hours (linear model, line R^2^>1.00). (B) Average maximum pupil constriction (488 nm) for 11 participants (±s.e.m) analysed as for Fig. 1A. Insets; Coloured lines show baseline pupil diameter (Fig. 1A) and maximum constriction of one participant (19,F) at circadian times of 3.5 h and 15.4 h (from Fig. 2A). Lines show linear regression; R^2^>0.47. (C) Average maximum pupil constriction (610 nm) for 11 participants (± s.e.m). Direct outer retinal cone photoreceptor contributions to the pupil do not vary diurnally (R^2^>0.64).

The diurnal response of the outer retinal cone photoreceptors was derived from the baseline pupil diameter and maximum pupil constriction. Figure 1A shows the average (n = 11 participants) baseline pupil diameter of the consensual eye (% baseline pupil diameter) each hour during the 24 h period, prior to stimulus onset during 10 s adaptation to the white, photopic fixation screen. Baseline pupil diameter did not vary significantly with circadian time (p = 0.668; mixed model univariate ANOVA). The slope of the best-fitting linear function was −0.02±0.39%.h^−1^. Baseline pupil diameter (in mm) varied significantly between participants (p<0.001; mixed model univariate ANOVA), consistent with a past report [12]. Figure 1B,C shows the cone contributions to maximum pupil constriction for the 488 nm and 610 nm stimuli. Maximum pupil constriction after light onset was best described by a linear function with circadian time (Fig. 1B,C). The average sample data revealed a small, albeit significant decrease in maximum pupil constriction at a rate of 0.012 mm.h^−1^ and 0.011 mm.h^−1^ for the 488 nm and the 610 nm lights respectively (p≤0.001; mixed model univariate ANOVA). Maximum pupil constriction decreased in 8/11 of participants for the 488 nm light and in 10/11 for the 610 nm light during the 24 h period. There was also a significant difference in the maximum pupil constriction (mm) between participants for both the 488 nm and 610 nm stimuli (p<0.001; mixed model univariate ANOVA).

### Circadian response of the ipRGC controlled post-illumination pupil response

**Figure 2:**
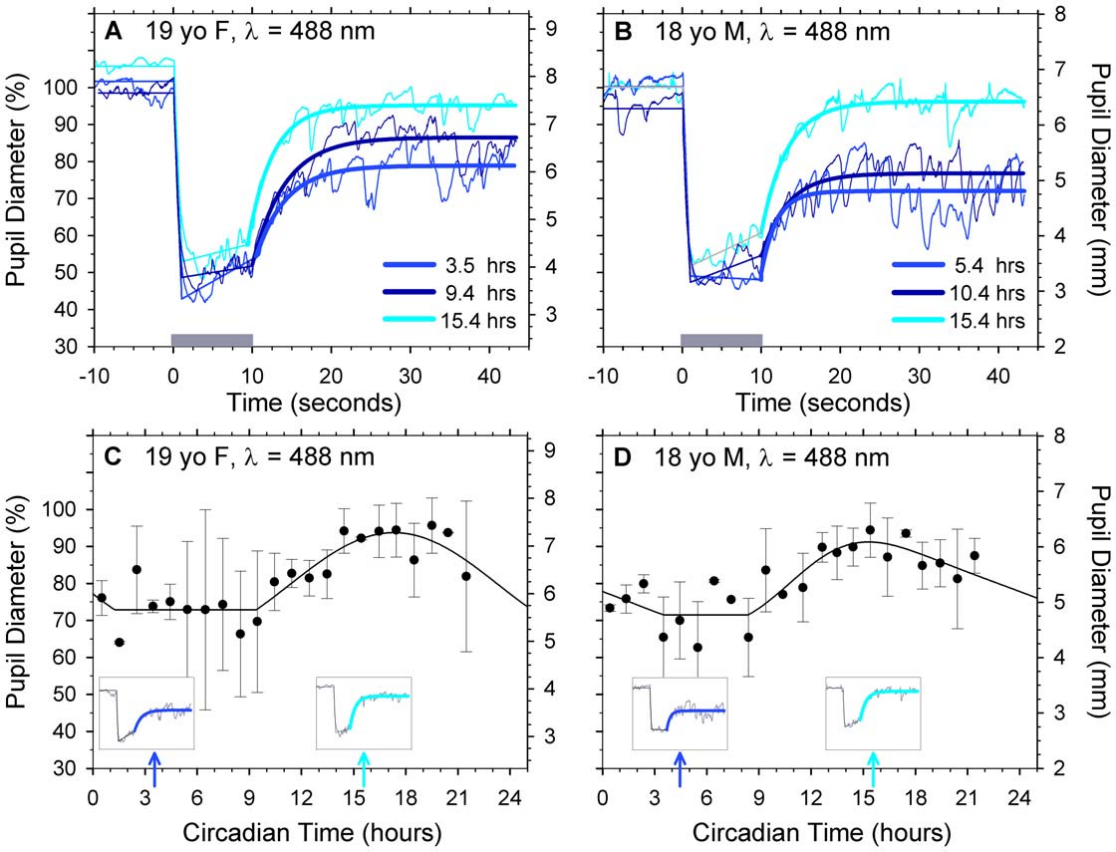
Circadian variation of the ipRGC controlled post-illumination pupil response of the pupil light reflex. Left and right columns show pupil light reflex data for two participants (19yo F, 18yo M). Left ordinates show pupil diameter (% baseline), right ordinates show pupil diameter (mm). (A) Post-illumination pupil responses at three circadian times were 75.0% (3.5 h), 83.2% (9.4 h) and 92.5% (15.4 h) of the mean baseline pupil diameter of 7.77 mm. The pupil light reflex (thin lines) was described by best-fitting linear and exponential functions (thick lines). (B) Post-illumination pupil responses of 71.4% (5.4 h), 77.0% (10.4 h) and 99.6% (15.4 h) of the mean baseline pupil diameter of 6.68 mm. (C) The post-illumination component of the pupil light reflex fitted with a skewed baseline cosine function (Eq 1) (data show mean ± s.d; filled circles; model, line) (R^2^ = 079). (D) As for panel (C) for participant 2 (18 yo M) (R^2^ = 0.71). Post-illumination pupil response (blue lines) from Figure 2B at 5.4 h and 15.4 h. Insets in panel (C) and (D) show the post-illumination pupil response (blue lines) from panel (A) at 3.5 and 15.4 h.

Figure 2A,B shows the pupil light reflex baseline diameter (pre-stimulus), response latency (delay in pupil constriction after light onset), maximum constriction and recovery (re-dilation after light offset) of two participants at three circadian times (488 nm stimulus light). Diurnal variation in the ipRGC response during a period of constant illumination manifests as a change in amplitude of the post-illumination pupil response (15–45 s). For participant 1 (19 yo F), the baseline post-illumination pupil response plateau was 5.83 mm at 3.5 h (25.0% constriction relative to baseline diameter), increasing to 7.18 mm at 15.4 h (7.5% relative constriction) (Figure 2A). A similar pattern was found for participant 2 (18 yo M) where the baseline post-illumination pupil response plateau was 6.68 mm at 5.4 h (28.6% constriction relative to baseline diameter), increasing to 6.65 mm at 15.4 h (∼0.4% relative constriction) (Figure 2B). Figure 2C shows the PIPR amplitude for participant 1 (mean ± s.d) as a function of circadian time and described by the best-fitting skewed baseline cosine function (Eq 1); initial PIPR amplitude was 72.9% of the baseline pupil diameter and increased to 93.7% at 17:19 h. Figure 2D shows the PIPR amplitude for participant 2 (mean ± s.d) as a function of circadian time and described by Eq 1; initial PIPR amplitude was 71.4% of the baseline diameter and increased to 99.6% at 17:19 h. This diurnal variation, independent of irradiance, in the intrinsic ipRGC post-illumination pupil response was demonstrated in all 11 participants with a mean baseline PIPR diameter of 82.5±7.8% and peak amplitude of 11.7±5.7% above the baseline PIPR value. Table 1 summarises individual participants post-illumination pupil response and melatonin values. In Table 1, the values are derived from the best-fitting skewed baseline cosine functions (Eq 1) to individual participant's diurnal PIPR and melatonin data, and all times are circadian.

**Table 1:** Post-illumination pupil response (488 nm) amplitude and timing derived from individual participant models (n = 11), melatonin onset and peak time.

*Participant (age, gender)*	*Baseline PIPR (%)*	*Minimum PIPR (%)*	*PIPR Difference (%)*	*PIPR amplitude decrease* * (h:min)	*PIPR amplitude minimum* (h:min)	*Melatonin peak* ** (h:min)
*30, F*	81.88	91.51	9.63	13:49	15:02	18:41
*31, F*	91.63	99.35	7.71	11:43	14:13	19:32
*19, F*	72.87	93.74	20.87	9:26	17:19	18:05
*27, F*	96.04	98.20	2.16	11:52	13:23	17:14
*27, F*	84.11	99.42	15.31	12:21	15:20	21:54
*30, F*	79.43	86.18	6.75	9:25	11:31	17:22
*24, M*	84.24	97.50	13.26	12:59	14:29	19:05
*21, F*	73.58	80.67	7.10	12:12	15:33	20:18
*18, M*	71.53	91.22	19.69	8:27	15:18	17:58
*26, M*	84.47	96.51	12.04	13:35	15:17	17:57
*24, M*	89.42	103.69	14.27	5:52	13:38	17:08

*Threshold decrease in ipRGC activity defined as 0.01 mm rise above baseline PIPR model value.

**Melatonin onset set to 14:00 h.

### Phase relationship between the ipRGC controlled human post-illumination pupil response and salivary melatonin

**Figure 3:**
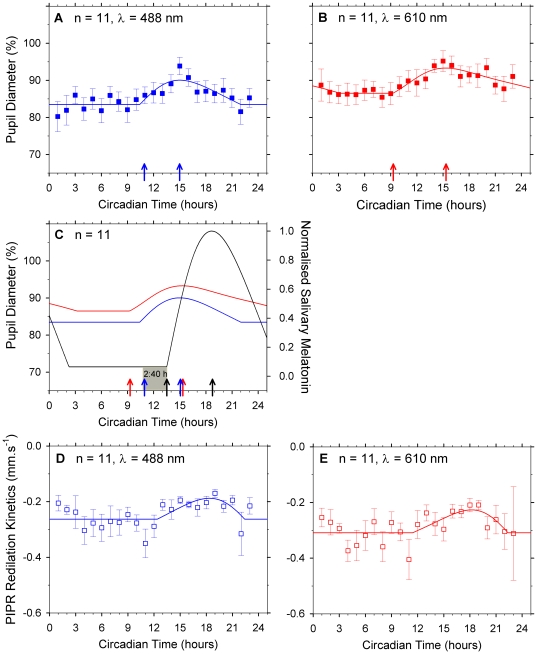
Comparison of the circadian response of intrinsic ipRGC and cone inputs to the post-illumination pupil response (PIPR) with salivary melatonin. Symbols (n = 11 participants; mean ± s.e.m) and lines (mean skewed baseline cosine function; Eq 1) encode intrinsic ipRGC (blue) and cone inputs (red) to the ipRGC controlled PIPR and salivary melatonin (black). Arrows indicate threshold change in activity based on the group model. (A) PIPR diameter (488 nm stimulus) began to increase at 10:50 h and peaked at 14:58 h (blue arrows) (R^2^ = 0.65). (B) PIPR diameter (688 nm stimulus) began to increase at 9:16 h and peaked at 15:18 h (red arrows) (R^2^ = 0.80). (C) Salivary melatonin began to increase at 13:30 h and peaked at 18:40 circadian hours (red arrows) (R^2^ = 0.96), 2:40 hours after PIPR (488 nm) began to increase. For the best-fitting salivary melatonin curve (Eq 1), *(b)* = 4.54 pM; peak amplitude above baseline *(H)* = 65.40 pM; width *(c)* = −0.091; phase *(Φ)* = 11.46 radians and skewness *(v)* = 0.302. (D) Re-dilation kinetics (mm.s^−1^) derived from the time-constant of the best-fitting exponential functions to the 488 nm PIPR. (E) Re-dilation kinetics (mm.s^−1^) for the 610 nm PIPR. Change in post-illumination pupil response amplitude and kinetics independent of the constant external illumination demonstrates circadian control of ipRGC activity.

Figure 3A,B shows the average post-illumination pupil response of the 11 participants (± s.e.m) with the 488 nm and the 610 nm lights as a function of circadian time and modelled (Eq 1) over 24 hours (R^2^ = 0.65). In this figure, circadian phase relative to clock time was determined by adjusting individual participant's DLMO to 14 h and grouping the PIPR data rounded to the nearest one hour bin. The intrinsic ipRGC (488 nm stimulus; Figure 3A) and cone-mediated ipRGC (610 nm stimulus; Figure 3b) response were derived from the average parameter values of the individual participants (Eq 1). Figure 3a shows that the post-illumination pupil response amplitude was maximal (largest % to baseline pupil diameter) at circadian times prior to 11:04±2:28 h. The PIPR amplitude then decreased until a minimum at 14:39±1.29 h (smallest % to baseline pupil diameter) and returned to the daytime baseline response at 23:50 h. Figure 3B shows that cone-mediated ipRGC response was maximal at circadian times prior to 11:41±1.41 h and was minimal at 14:53±1:37 h. The ipRGC controlled pupil response shows significant dynamic circadian changes under controlled illumination and stimulus irradiance for both 488 nm and 610 nm stimuli (p<0.001; mixed model univariate ANOVA). Tukey post-hoc analyses indicated the PIPR is significantly different at 15 h compared to other circadian times for the 488 nm (p<0.05 for the circadian hours: 2–13, 17–24 h) and 610 nm lights (p<0.05 for the circadian hours: 2–12, 17, 18, 20 and 24 h). The PIPR also showed significant variation between the individual participants (p<0.001; mixed model univariate ANOVA).

Figure 3C shows the phase position of the SCN and retina by comparing the circadian variation in the average (n = 11 participants) melatonin concentration (black line; right ordinate normalised to one), intrinsic ipRGC post-illumination pupil response (blue line) and cone inputs to the ipRGC post-illumination pupil response (red line). The change in ipRGC driven PIPR (Fig. 3C, blue line) is in temporal phase advance of the SCN (Fig. 3C, black line), preceding the onset of melatonin secretion in saliva production by 2:40 h. The return of the PIPR to baseline at 23:50 h shows a 2:26 h temporal phase advance with melatonin offset at 26:16 h. Although ipRGC long-wavelength sensitivity is >2 log units less than that of the cones photoreceptors at 610 nm [23], cone contributions to the post-illumination pupil response (Fig. 3, red line) measured with the 610 nm light demonstrates similar temporal circadian synchrony to the intrinsic ipRGC response.

Figure 3D,E shows the average (± s.e.m) redilation kinetics of the PIPR (exponential time-constant) with the 488 nm and the 610 nm lights (n = 11 participants) as a function of circadian time and modelled (Eq 1) over 24 hours with the skewed baseline cosine function (R^2^ = >0.38). The kinetics of the 488 nm PIPR redilation show significant variation with circadian time (p = 0.014; mixed model univariate ANOVA), similar to the PIPR amplitude data (Fig. 3A), with the slowest kinetics at 19 h. Tukey post-hoc analyses indicated the redilation kinetics at 19 h are significantly different compared to other baseline circadian times (p<0.05 for circadian hours: 5, 6 7, 11, 12, 22 h). The kinetics of the 610 nm PIPR redilation data showed a similar trend to the 488 nm data but this was not significant (p = 0.124) possibly due to the increased variation.

## Discussion

This study addressed pending questions related to intrinsically photosensitive retinal ganglion cells (ipRGC) properties [30] and demonstrated that basic functional retinal outputs, namely the intrinsic ipRGC post-illumination pupil response (PIPR) and cone networks driving ipRGCs inputs to the pupil, are subject to circadian variation *in vivo*. We established that the change in ipRGC controlled post-illumination pupil response is on average, 2:40 h in advance of the onset of melatonin secretion, and the minimum post-illumination pupil response occurs on average, 1.31 h after melatonin onset (Figure 3). This minimum occurs with a slowing of the intrinsic post-illumination pupil response redilation kinetics. In contrast to other expressions of circadian rhythm, such as cognitive throughput [31], the ipRGC's also act as transducers for input to the circadian system. Outer retinal cone inputs to the pupil light reflex however, do not demonstrate a circadian variation over the 24 period. The mechanisms attenuating ipRGC-mediated function may be a driver for maintaining normal sleep/wake patterns in geographical areas exposed to extreme dark/light cycles.

The amplitude and timing of the ipRGC controlled post-illumination pupil response relative to salivary melatonin shows individual differences (Table 1). There is evidence for multiple sources of inter-individual variability in both the pupil light reflex [12], and in melatonin expression [32]. In this study, the baseline PIPR amplitudes of the participants (Table 1) were within the range of reported values [23], [26]. Likewise, variation in melatonin concentration and temporal duration was also consistent with past reports [33]. The variation in the ipRGC controlled post-illumination pupil response is unlikely to be due to the accumulating effects of sleepiness. Converse to the data (Figure 1), sleepiness decreases pupil diameter and increases pupil fluctuations [16], [34], [35], [36]. The inflection in the PLR occurred several hours before the habitual bedtime of each participant, a period of increased alertness [37], and the participants had not been sleep-deprived prior to the study. The homeostatic component of sleep propensity predicts increasing sleepiness during wake [38], and recovery of the PLR would not be expected. While further investigations will be required to identify the sources of inter-individual variation in amplitude and timing of the diurnal post-illumination pupil response function, the general relationship between this response and the DLMO was consistent across all observers.

The experimental paradigm differentiated inner and outer retinal function based on their contributions to the pupil light reflex. Baseline pupil diameter can reflect rod, cone and ipRGC input depending on the stimulus spectral distribution, irradiance and duration [39]. For the photopic fixation screen used in the pupillometer, the contribution of cone photoreceptors to baseline pupil diameter is largest during the first 10 seconds of pre-stimulus recording, before receiving additional contributions from ipRGCs [4], [39]. Although rod photoreceptors drive photoentrainment at scotopic and photopic light levels via ipRGC retinal circuitry [5] and contribute significantly to the steady-state pupillary diameter [39], evidence suggests that baseline diameter shows diurnal variation only when rods are active [40]. As such, the light levels in this study minimized rod contributions to the pupil light reflex and no diurnal variation in baseline pupil diameter was observed (Figure 1A). The pupil constriction latency at light onset depends on the temporal dynamics of cone, iris muscle and pupil innervation pathways, but not ipRGCs (latency >1.78 s) [39]. The data show that outer-retinal rod and cone photoreceptor contributions to the pupil light reflex do not show circadian variation but a linear decrease. The linear decrease in maximum pupil constriction diameter during the diurnal testing (Figure 1B,C) may reflect cortical inhibition of the parasympathetic pathway at the Edinger-Westphal nucleus due to the cumulative cognitive demand [41] of the 20–24 hour testing.

Contrary to *in vitro* electrophysiological data in rat retinal preparations [30], we show that human ipRGC-mediated function varies diurnally, and is in phase advance of melatonin when studied *in vivo* under conditions of constant illumination and stimulus irradiance. The temporal phase of ipRGC-mediated signalling and salivary melatonin onset may depend on the timing and action of neuromodulators in the photoneuroendocrine pathway, variables that are yet to be defined. The diurnal reduction in PIPR amplitude and slowing of the redilation kinetics point to an evening central gating of ipRGC or of post-retinal ipRGC signalling prior to SCN-mediated melatonin release, suggesting a resonant network between central and peripheral clocks, possibly via clock gene feedback loops [42]. This might include negative feedback loops that modulate the amplitude of peripheral signals independently of environmental light level. Negative feedback loops have been shown to oscillate gene expression and protein concentration [43] and ipRGCs demonstrate circadian variation in both melanopsin mRNA and protein synthesis [44]. An implication of our data is that changes in SCN sensitivity to external light, demonstrated by the phase-response curve [45], may be at least partly mediated by these pathways, and provide a starting point for understanding the time course of feedback mechanisms gating ipRGC activity and their role in circadian phase shifting.

## Methods

### Participants and Ethics Statement

Eleven participants (4 Male, 7 Female: age range 18–30 years; mean ± s.d = 25.67±4.21 years) were recruited in accordance with Institutional Ethics Requirements and the tenets of the Declaration of Helsinki. The Queensland University of Technology Human Research Ethics Committee approved this study (#0800000546). Written informed consent was obtained after the purpose and possible risks of the experiment were explained. Because the presence of a circadian disorder may be associated with abnormal retinal circadian rhythms only participants with robust, normal circadian rhythms were included in this study. Absence of medical, ocular, sleep and circadian disorders was determined by medical examination, case history, Pittsburgh Sleep Quality Index questionnaire [46], one-week assessment of habitual sleep and wake with Actigraphy (Actiwatch 2, Phillips) and a sleep diary. Participants were non-smokers, moderate caffeine consumers (<4 beverages/day), did not use sleep medications, had not crossed more than one time zone in the month prior to testing and were not shift-workers.

Normal vision was determined by ophthalmological examination according to the following criteria: no retinal or optic nerve disease (ophthalmoscopy and fundus photography), Bailey-Lovie LogMAR visual acuity ≥6/6 (participants mean left eye subjective refraction = −0.36±0.92/−0.25±0.32 dioptres), trichromatic colour vision (HRR Pseudoisochromatic plates; Lanthony desaturated D-15), stereo acuity <60″ arc (Titmus stereo test), Pelli-Robison contrast sensitivity ≥1.75 and intraocular pressure ≤21 mm Hg (I-care tonometer). Lenses were graded using a Nikon photo slit lamp for cortical, nuclear and posterior subcapsular cataract and all participants had normal lenses for their age (Grade<1) [47].

To assess subjective sleep quality during the week prior to testing [46], participants were screened with the Pittsburgh Sleep Quality Index (PSQI). All participants were determined to have normal sleep quality (PSQI mean±s.d; 3.3±1.3). To subjectively determine participants habitual sleep patterns and wake time, the Pittsburgh Sleep Diary (PghSD) was recorded at bedtime and at wake time one week prior to testing [48]. Participants recorded a subjective mean wake time of 7:55 am±0:54 min and sleep time of 11:59 pm±0:28 min.

To objectively record participant's habitual sleep patterns and wake time [49], actigraphy (wrist worn AW-L Actiwatch; Phillips Respironics, Bend, Oregon 97701 USA) measured participants' motor activity and light exposure (range: 0.4–150000 lux) every minute for one week prior to testing. Rest/sleep intervals were estimated using Actiware 5.2 software (Philips Respironics, Bend, Oregon 97701 USA). The mean actigraphic wake time of the 11 participants was 8:00 am±1:14 h and sleep time of 11:54 pm±0:48 h.

### Apparatus: Pupillometer

The consensual pupil light reflex of the left eye was recorded with an infrared Pixelink camera (IEEE-1394, PL-A741 FireWire; 480×640 pixels; 62 frames.s^−1^) through a telecentric lens (Computar 2/3″ 55 mm and 2× Extender C-Mount) in response to a calibrated, monochromatic (488 nm, 610 nm; 10–12 nm full-width at half maximum, Edmund Optics) 14.2 log photon.cm^−2^.s^−1^, 10 s, 7.15° stimulus presented to the right eye (dilated and cyclopleged) using a Maxwellian view optical system, controlled and analysed with custom software (MatLab, Mathworks) [11]. The viewing distance (1.15 m) of the 6.3°×8.9° photopic back-lit fixation screen (116 cd.m^−2^) for the right eye was determined by control study to minimize accommodation and convergence driven pupil fluctuations. Temple bars, head restraint and chin rest stabilised head position in the pupillometer. The measured spectral sensitivity of the post-illumination pupil response (peak ∼483 nm) confirmed our technique as a direct measure of ipRGC function [11].

**Figure 4:**
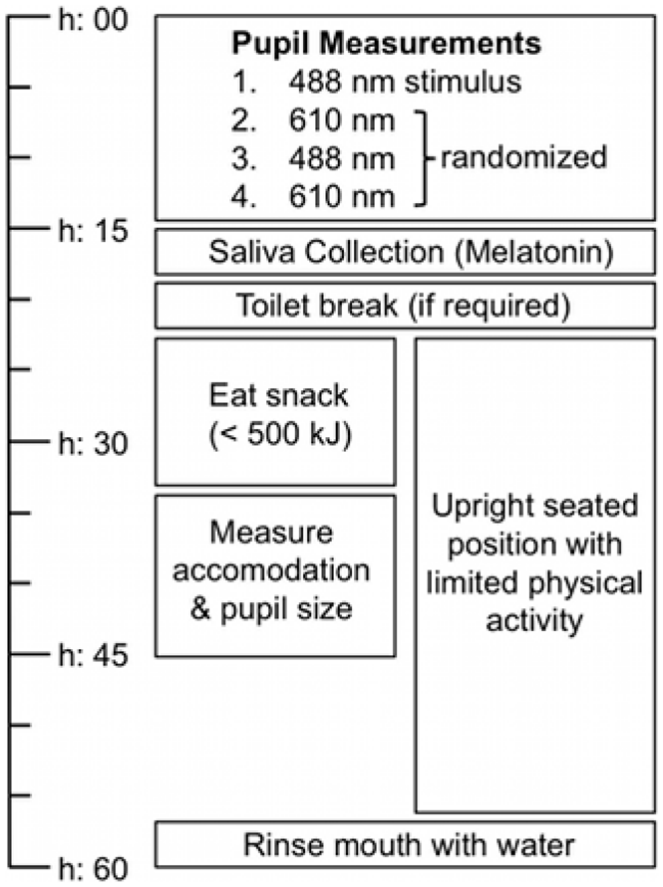
Circadian testing protocol. The flowchart identifies the order of measurements conducted each hour during the 24 h test period.

The pupil light reflex was determined by four consensual pupil recordings of 55 seconds (10 seconds pre-stimulus, 10 seconds stimulus and 35 seconds post-stimulus) repeated every hour (Figure 4). The short wavelength stimulus (488 nm) was chosen to maximize ipRGC contributions to the PIPR [11], [23] and the long wavelength stimulus (610 nm) was chosen to study outer retina cone contributions to the PIPR and as a control of non-specific factors such as fatigue on the PIPR [11], [23], [26]. The maximum constriction amplitude represents the cone contribution alone to the pupil light reflex [11] because ipRGC latency is >1.78 s for both stimulus lights [39].

### Procedure

The circadian response of ipRGCs and cone photoreceptors were determined during a 20–24 h laboratory test period, during which the participant remained awake. On the day of testing, participants arrived at the laboratory at 8 am for set-up and alignment in the pupillometer, and rinsed their mouth with water in preparation for the first salivary collection, prior to the commencement of the first pupil measurements at 9 am (Figure 4). To maximise pupil diameter (>6.5 mm), control retinal illumination and minimise the effects of accommodation on pupil diameter, the participant's right pupil was cyclopleged with 1.0% cyclopentolate. Subjective accommodation was assessed using an optometer (Hartinger, Rodenstock) and cyclopentolate was re-instilled as required. Exogenous circadian cues of activity (minimum), sleep (none), posture (seated upright), caffeine (none), ambient temperature (23–25°C), caloric intake (aliquots <500 kJ.hr^−1^) and ambient illumination (10 lux) [50] were controlled for the entire test duration.

Figure 4 is a flowchart timeline of the hourly measurements procedures. At the start of each hour, after alignment in the pupillometer, four pupil light reflex measurements were recorded (2×488 nm; 2×610 nm). Salivary sample collection for dim light melatonin onset (DLMO) was then completed according to standard protocols [51]. In between measurements, participants remained in an upright-seated position with limited physical activity as monitored by the actigraph. The constant laboratory illumination (10 lux) and repeated hourly delivery of an equivalent stimulus energy for the pupil light reflex measurements allowed us to determine if circadian variation in ipRGC and cone photoreceptor activity was independent of environmental light.

### Melatonin Assay

The circadian phase of the suprachiasmatic nucleus was inferred from the melatonin circadian rhythm extracted from salivary samples collected after completion of each hourly pupil recordings. DMLO saliva collection protocols were followed [51], [52]. Salivary samples were collected after participants gently chewed on a cotton swab (Salivettes; Sarstedt, Nümbrecht, Germany) for 2 minutes. Participants rinsed their mouths 15 minutes prior to sample collection (Figure 4) and were required to refrain from brushing their teeth during the test period. Saliva samples were centrifuged (3 min, 3000 rpm; Hettich Universal 320 centrifuge) and stored at −80°C within 24 hours of collection, before being shipped on dry ice to the Circadian Physiology Group at the University of Adelaide Medical School for analysis. Melatonin levels were determined by radioimmunoassay (sensitivity <4.3 pM) using the methods described by Voultsios, Kennaway and Dawson [51] using Bühlmann Laboratories assay reagents (Schönenbuch, Switzerland). Dim-light melatonin onset was defined as a rise of 0.01 pM in salivary melatonin. The salivary melatonin assay has sensitivity and accuracy comparable with mass spectrometry assay of plasma melatonin [51].

### Data Analysis

A simple linear and exponential model described the pupil light reflex. The baseline pupil diameter (cone and ipRGC activity), maximum pupil constriction (cone) and post-illumination pupil response amplitude (intrinsic ipRGC; cone inputs to ipRGCs) were derived from the best-fitting model parameters and used for statistical analysis.

Circadian phase was estimated by modelling the individual participants 24 h melatonin data as a function of time with the skewed baseline cosine function (SBCF) described by Van Someren and Nagtegaal [53] where,
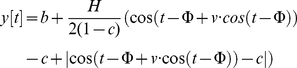
(1)and *b* is baseline salivary melatonin, *H* is the amplitude above baseline, *c* is width, Φ is phase and *v* is skewness. Time *t* is in radians (0–2π) representing 0–24 h. With this model, the melatonin peak is defined as the time (radians) when the modelled melatonin value (*y*) is equal to the sum of baseline melatonin (*b*) and the amplitude (*H*). Each participant's DLMO was defined as the time (*t*) when the individual modelled melatonin first increased by 0.01 pM above baseline. The melatonin offset time was defined as the time (*t*) after the melatonin peak when the individual modelled melatonin was 0.01 pM above the baseline value. Parameter optimization was achieved by floating all parameters and minimizing the sum-of-squares differences between the data and free parameters using the solver module of an Excel spreadsheet. Once individual DLMO, peak melatonin and offset times were calculated, DLMO time was used to align the circadian phase of all participants. Because DLMO clock time varies between participants, individual melatonin onset values were arbitrarily set to 14 h to adjust clock time to circadian time.

Statistical analysis used a Linear Mixed Model (random effects) univariate ANOVA to determine if the pupil light reflex components varied significantly with circadian time. The Linear Mixed Model is designed for analysis of unbalanced repeated measures, and can accept missing values without excluding entire sections of data. The hypothesis of the Linear Mixed Model was that the dependent variable (baseline pupil diameter, maximum constriction, PIPR) is not significantly different when the factor (time, repeat) was varied. A *p*<0.05 was considered statistically significant.
